# Compendium Der Neueren Medicinischen Wissenschaften

**Published:** 1875-05-15

**Authors:** 


COMPENDIUM DER NEUEREN MEDICINISCHEN WISSENSCHAFTEN. Von Bernhard Kraus. (*A compend of the more recent medical sciences, etc.*)

This is the title of a neat volume of about 800 pages, just sent us across the ocean by the author, the well known editor of the *Allg. Wiener Med. Zeitung.*

Without claiming to announce any new facts, the author states his purpose to be to present the recent advances in medicine in a condensed and practical shape to the reader who lacks time or means to study the special treatises on these subjects. With this apology on his part, he has nevertheless treated the different departments into which the work is divided with such completeness that they command an authoritative consideration.

Briefly glancing over the work, we must especially recommend the first department, Clinical Thermometry, for the precision and thoroughness which it possesses. In the next chapter, Clinical Sphygmometry, we notice, however, that the researches and instrument of Marey, the practical founder of the science, are almost entirely ignored—certainly not from want of acquaintance with the literature of the French, as the bibliographical index at the end of each chapter shows the thorough command of the same. On the other hand, English, and perhaps also American, authorities receive less consideration than they deserve. To the other chapter—consisting of Auscultation and Percussion, Ophthalmoscopy, Microscopy, Urinalysis, Laryngoscopy, Anomalies of Speech, Otology, Electrology, Hygiene, and Toxicology—similar remarks are applicable. While the use of observations with the ophthalmoscope are very carefully treated, we find very little reference to its application in nervous diseases. The chapter on microscopy is written in such a concise and practical manner as to deserve special mention. The uses of electricity, etc., are also described in a similar manner. The department of anomalies of speech would undoubtedly be of particular interest to the American reader on account of its neglect in this country. In some parts of the work a little more conciseness might be desirable, as explanatory remarks occasionally seem to exceed their limits of space. This fault is less noticeable in parts written by the co-operators of the author.

But these defects are of such minor consideration that we state it as our opinion unhesitatingly that, notwithstanding our numerous *vade mecums* etc., a translation of this work would prove a highly valuable addition to American literature. H. G.

